# Geographical Variation of Diet Composition of *Cervus nippon kopschi* in Jiangxi, China Based on DNA Metabarcoding

**DOI:** 10.3390/ani15070940

**Published:** 2025-03-25

**Authors:** Xiao Sun, Feiyan Lv, Xueqin Hu, Jun Tian, Ruijie Yang, Jie Yao, Zhiqiang Huang, Jiancheng Zhai

**Affiliations:** 1 Natural Reserve Planning and Research Institute, East China University of Technology, Nanchang 330013, China; 2 School of Earth Sciences, East China University of Technology, Nanchang 330013, China; 3 College of Animal Science and Technology, Jiangxi Agricultural University, Nanchang 330029, China

**Keywords:** diet composition, *Cervus nippon kopschi*, DNA metabarcoding, geographical variation, Taohongling Sika Deer National Nature Reserve

## Abstract

Food resources are the fundamental basis for the survival and reproduction of animals. Diet research is the foundation for understanding their ecological habits and is of great significance for evaluating their survival status and carrying out effective protection and management. South China sika deer (*Cervus nippon kopschi*) is the most endangered subspecies of wild sika deer in China, with a small population and a shrinking distribution area. Here, we used DNA metabarcoding technology to study the diet composition of *C. n. kopschi* in Taohongling Sika Deer National Nature Reserve in Jiangxi, China. Comparative analysis of diet composition among different areas (A–E) of *C. n. kopschi* was conducted, as well as *C. n. hortulorum* raised in the same areas. We found that the dominant families in the diet composition of *C. n. kopschi* were Rosaceae (46.73% of relative abundance), Anacardiaceae (6.02%), Poaceae (5.54%), and Fabaceae (3.92%), with *Rubus* (45.43%) being the absolute dominant genus. Thirty-two preferred plant species were identified in the diet of *C. n. kopschi*. The highest relative abundance (45%) was *Rubus reflexus*. The dominant family and genus in the diet composition of *C. n. hortulorum* were Fabaceae (33.89%) and *Pueraria* (32.87%), respectively. Of the 15 preferred plant species, the highest relative abundance was *Pueraria montana* (33%). The richness, diversity, and evenness of diet composition in Area B of *C. n. kopschi* were the highest among all areas, with significant differences compared to Areas C, D, and E. Diet composition of deer in Area A did not differ significantly from those in other areas. Principal coordinate analysis (PCoA) and nonmetric multidimensional scaling (NMDS) both indicated a significant separation in the diet composition of *C. n. kopschi* and *C. n. hortulorum*, while Area B showed significant separation from the other areas. This study elaborates on the diet composition information of *C. n. kopschi* and can provide a reference for the protection and improvement of the habitat of sika deer.

## Simple Summary

Food resources are the fundamental basis for the survival and reproduction of animals. Diet research is the foundation for understanding their ecological habits and is of great significance for evaluating their survival status and carrying out effective protection and management. South China sika deer (*Cervus nippon kopschi*) is the most endangered subspecies of wild sika deer in China, with a small population and a shrinking distribution area. We used DNA metabarcoding technology to study the diet composition of *C. n. kopschi* in Taohongling Sika Deer National Nature Reserve in Jiangxi, China. Comparative analysis of diet composition among different areas of *C. n. kopschi* was conducted, as well as *C. n. hortulorum* raised in the same areas. We found that the dominant families in the diet composition of *C. n. kopschi* were Rosaceae, Anacardiaceae, Poaceae, and Fabaceae, with *Rubus* being the absolute dominant genus. The dominant family and genus in the diet composition of *C. n. hortulorum* were Fabaceae and *Pueraria*. Thirty-two and fifteen preferred plant species were identified in the diets of *C. n. kopschi* and *C. n. hortulorum*. This study elaborates on the diet composition information of *C. n. kopschi* and can provide a reference for the protection and improvement of the habitat of sika deer.

## 1. Introduction

Sika deer (*Cervus nippon*) were widely distributed in eastern Asia, including China, Japan, South Korea, and North Korea, as well as northern Vietnam and the Primorsky Krai of Russia, with a total of 13 subspecies recorded [1]. There are six subspecies of sika deer in China’s history, namely *C. n. hortulorum*, *C. n. sichuanicus*, *C. n. kopschi*, *C. n. grassianus*, *C. n. mandarinus*, and *C. n. taiouanus* [2]. However, hunting and habitat loss have led to the extinction or risk of extinction of wild populations of sika deer. At present, the wild populations of sika deer with confirmed distribution in China include *C. n. sichuanicus* and *C. n. kopschi*. There is still controversy over the existence of wild populations of *C. n. hortulorum*, while the other three subspecies have become extinct in the wild [3]. Sika deer have been listed as a national first-class protected animal in China and also as an endangered animal in China’s Red List of Endangered Animals. *C. n. kopschi* is the most endangered subspecies of wild sika deer in China, with a small population and a shrinking distribution area [4]. It is mainly distributed in the northeast of Jiangxi Province, northwest of Zhejiang Province, and southern Anhui Province, with significant barriers between different distribution areas. Taohongling Sika Deer National Nature Reserve in Jiangxi Province is one of the most concentrated and important distribution areas of *C. n. kopschi*, with the largest existing population. According to reports, the population of *C. n. kopschi* in the Nature Reserve has gradually increased from 60 in 1983 to 624 in 2021, a tenfold increase [5,6]. However, it has been found that some individuals of sika deer have spread outside the Nature Reserve, and the plant community has gradually undergone a succession from grassland- and shrub-dominated to shrub- and tree-dominated. Sika deer belong to forest-edge animals and prefer grasslands and shrubs. Therefore, these changes are not conducive to the survival and protection of the *C. n. kopschi* population.

Food resources are the material basis on which animals rely for survival and reproduction. Diet research is the foundation for understanding animal ecological habits and is important for conducting research on foraging strategies, interspecific relationships, food web structures, ecological niches, etc. It is of great significance for evaluating the survival status of rare and endangered animals and carrying out effective protection and management [7,8,9,10]. Traditional dietary research mainly uses microscopic morphological comparison analysis of undigested food residues in animal feces to obtain diet composition information [11,12]. However, there are many limitations to this method, such as high requirements for researchers’ identification and recognition abilities, significant subjective factors, cumbersome processes, and difficulty in ensuring accuracy and reproducibility [11,13]. With the development of molecular biology, high-throughput sequencing and DNA metabarcoding technology have provided a more efficient and accurate method for dietary research [12,14,15]. Based on the availability of species sequences in the database used for identification, DNA meta barcodes can accurately identify amplified DNA sequences. In ungulates, an average of 90% of DNA sequences of plant material found in fecal pellets were identified at the genus or species level, while 75% of plant fragments were detected using microscopy. For example, major dietary components were four times more likely to be detected by DNA than by microscopic examination in wild boar (*Sus scrofa*) [16]. DNA metabarcoding has been successfully applied to carnivores and herbivores such as the leopard cat (*Prionailurus bengalensis*), Asiatic golden cat (*Catopuma temminckii*) [17], gray wolf (*Canis lupus*), Eurasian lynx (*Lynx lynx*) [18], Eurasian otter (*Lutra lutra*) [19], black-throated blue warbler (*Setophaga caerulescens*) [20], black-necked crane (*Grus nigricollis*) [21], roe deer (*Capreolus capreolus*), fallow deer (*Cervus dama*) [22], European bison (*Bison bonasus*) [23], and other herbivores [24].

Understanding the dietary habits of key or flagship species within nature reserves, as well as their relationship with forest stands, is important for designing reliable conservation strategies [25,26,27,28]. DNA metabarcoding technology of animal fecal samples has been proven to be a useful and rapid tool for estimating the diet of herbivorous animals [27,29]. In this study, we investigated the diet composition of sika deer based on DNA metabarcoding. We aimed to determine the (1) diet composition of *C. n. kopschi* in the Taohongling Sika Deer National Nature Reserve, (2) differences in diet composition of *C. n. kopschi* in different areas of the nature reserve, and (3) differences in diet composition between *C. n. kopschi* and *C. n. hortulorum* raised on an enclosed farm within the nature reserve. This study elucidates the diet composition information of sika deer, which can provide a reference for the improvement and protection of the habitat of *C. n. kopschi* and provide a scientific basis for the development of effective protection measures.

## 2. Materials and Methods

### 2.1. Study Area

**Figure 1:**
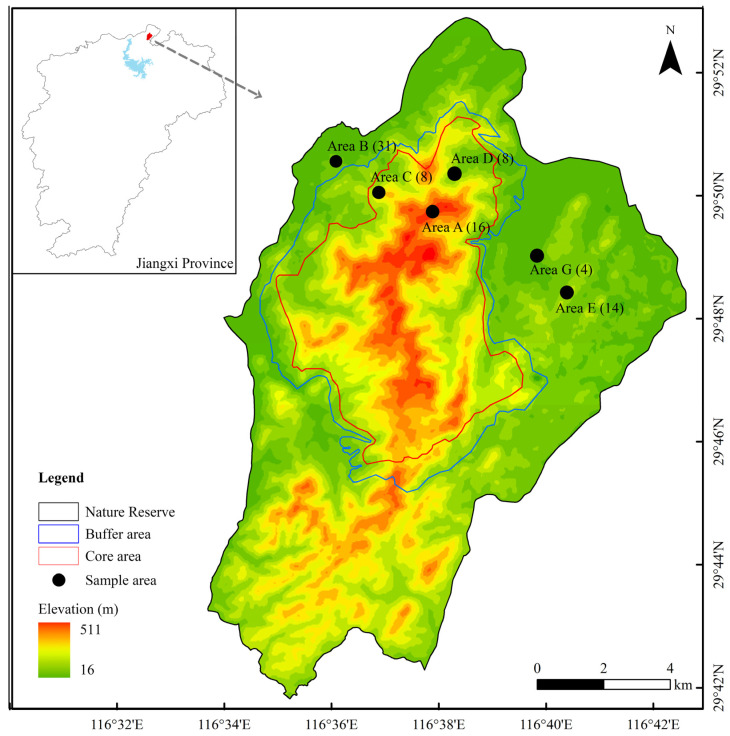
Sampling locations in Taohongling Sika Deer National Nature Reserve. The numbers in parentheses indicate the corresponding sample sizes. The blue area in the upper left corner represents Poyang Lake.

Taohongling Sika Deer National Nature Reserve in Jiangxi Province is located in Pengze County, Jiujiang City, on the south bank of the middle and lower reaches of the Yangtze River (116°32′–116°43′ E, 29°42′–29°53′ N) (Figure 1). The eastern, northern, and western sides of the nature reserve are bounded by highways, while the southern side has a more complex terrain and is bounded by valleys. The total area is 12,500 hectares, with a maximum length of 18.25 km from north to south and a maximum width of 13.4 km from east to west. The nature reserve is unfenced, and deer can move freely into and out of the reserve. The terrain is composed of gently undulating low mountains and hills, with the highest elevation being 536.6 m. The climate belongs to a warm and humid monsoon climate, with obvious seasonal changes. The annual average temperature is 16.5 °C, and the average annual precipitation is 1172 mm. The vegetation composition is complex, divided into 7 vegetation types and 17 formations. The vegetation types mainly include warm coniferous forests, deciduous broad-leaved forests, evergreen deciduous broad-leaved mixed forests, bamboo forests, deciduous broad-leaved shrubs, evergreen broad-leaved shrubs, and shrub grassland.

### 2.2. Sample Collection

Based on historical survey data and infrared camera monitoring information of Taohongling Sika Deer National Nature Reserve, five distribution areas of *C. n. kopschi*, namely Area A, Area B, Area C, Area D, and Area E, were selected (Figure 1). The habitat type of Area A is shrub grassland, and the dominant species are *Pueraria lobata* and *Boehmeria nivea*. Area B is a nursery base with a variety of nursery plants, such as *Morus alba*, *Rhus chinensis*, *Prunus persica*, *Ligustrum lucidum*, *Heptapleurum heptaphyllum*, etc. The habitat type of Area C is deciduous broad-leaved forest, and the dominant species are *Loropetalum chinense* and *Quercus dentata*. The habitat type of Area D is deciduous broad-leaved shrub, and the dominant species is *Acer miyabei*. The habitat type of Area E is evergreen broad-leaved shrub, and the dominant species are *Cunninghamia lanceolata* and *Rubus palmatus*. Within the distribution areas of *C. n. kopschi*, high-frequency activity areas such as animal trails and ridges were chosen. Randomly placed line transects were used to collect fecal pellets. The research team conducted a survey in the five distribution areas of *C. n. kopschi* in July 2022. Fresh feces of *C. n. kopschi* were collected using disposable gloves, and individual numbers, coordinate points, and dates were recorded in detail. They were temporarily stored in a portable low-temperature refrigerator and immediately stored in a −20 °C freezer upon return to the nature reserve management station. After transportation to the laboratory, they were frozen at low temperature until experimental analysis. A total of 77 fecal samples of *C. n. kopschi* were collected. In addition, the research team collected a total of 4 fecal samples of *C. n. hortulorum* (Area G) raised on an enclosed farm within the nature reserve.

### 2.3. DNA Processing and High-Throughput Sequencing

The genomic DNA were extracted from fecal samples of sika deer by MagBeads FastDNA Kit (MP Biomedicals, Santa Ana, CA, USA), quantified by Nanodrop (Thermo Fisher Scientific, Waltham, MA, USA) and detected by 1% agarose gel electrophoresis for DNA quality, and they were then frozen. The *rbcL* primers were used for PCR amplification. The upstream primer is 5′-ATGTCACCACCAACAGAGACTAAAGC-3′, and the downstream primer is 5′-CGTCCTTTGTAACGATCAAG-3′ [30]. PCR cycling was performed by initial denaturation at 98 °C for 2 min, followed by 30 cycles of 98 °C for 15 s, 50 °C for 30 s, and 72 °C for 30 s, and a final extension of 72 °C was performed for 5 min. The PCR amplification products were purified and recovered using magnetic beads. Both DNA extraction and PCR amplification were performed using double-distilled water as the negative control to check for contaminations. Fluorescence quantification of PCR-amplified recovered products was performed using a Microplate reader (BioTek Inc., Winooski, VT, USA). According to the fluorescence quantification results, each sample was mixed in the corresponding proportion according to the sequencing requirements of each sample. A sequencing library was prepared using the TruSeq Nano DNA LT Library Prep Kit (Illumina, San Diego, CA, USA), followed by paired-end sequencing using a MiSeq sequencer. Sequencing length is 230 bp.

### 2.4. Bioinformatics and Statistics Analysis

Bioinformatics analysis was conducted using Quantitative Insights into Microbial Ecology 2 (QIIME 2, 2019.4) [31]. The fastq_filter plugin of Vsearch effectively removed primers and performed quality control on sequence data. The derep plugin was used to eliminate duplicate and low-abundance sequences. Using the cluster_size plugin, we clustered sequences with 97% similarity and selected the most abundant or central representative operational taxonomic units (OTUs) [32]. Chimeric sequences were removed through self-alignment or database-based methods. The global search function of Vsearch enabled us to compare OTU sequences with databases, thereby obtaining species information for each sample. Nucleotide sequences were aligned in the National Center for Biotechnology Information (NCBI) database using the Basic Local Alignment Search Tool (BLAST). Then, the BROCC algorithm (https://github.com/kylebittinger/q2-brocc#the-brocc-algorithm) (accessed on 1 July, 2024) was used for the above OTU annotations [33]. Blastn was used to compare data with the Nucleotide Sequence Database to match the most similar species, with a sequence similarity threshold of 97%, and then species classification annotations were performed on each taxonomic unit. Subsequent analysis only retained OTUs that might serve as deer’s food and removed sequences from the bacteria phylum and those not assigned to the phylum. Due to the fact that samples with food group reads less than 100 were generally considered amplification failures, food group reads less than 100 were removed. Meanwhile, OTUs with a read count of less than 1% in each sample were also excluded from subsequent analysis to avoid false positive results. Relative abundance is the percentage of samples that a taxonomic group is detected in.

Alpha diversity (species diversity within sample areas) was comprehensively evaluated using QIIME 2 (2019.4) [31], including the Chao1 index, observed species index [34], Shannon index [35], Simpson index [36], Pielou’s evenness index [37], and Good’s coverage index [38]. Box plots were drawn using R software to visually display the differences in alpha diversity between different areas, and Kruskal–Wallis’s rank sum test and Dunn’s test were used as post hoc tests to verify the significance of the differences. Principal coordinate analysis (PCoA) and nonmetric multidimensional scaling (NMDS) were used to analyze the beta diversity (species diversity among areas) of diet composition in different areas. Cluster analysis was used based on the unweighted pair group method with arithmetic means (UPGMA). Analysis of Similarities (ANOSIM) and Adonis Analysis (Permutation Multivariate ANOVA) were used to test the differences in diet composition among different areas. To identify the differences in diet composition among areas, linear discriminant analysis (LEfSe, LDA = 2.0) was performed on the relative abundance of food sequences in different areas. The statistical analysis was conducted using SPSS v.18.0 (SPSS Inc., Chicago, IL, USA). The mean and standard deviation of data were computed, and Duncan’s test (*p* < 0.05) was used to indicate a significant difference. Analysis of Variance was used to test for significant differences between parameters and samples.

## 3. Results

### 3.1. Analysis of Sequencing Results

**Figure 2:**
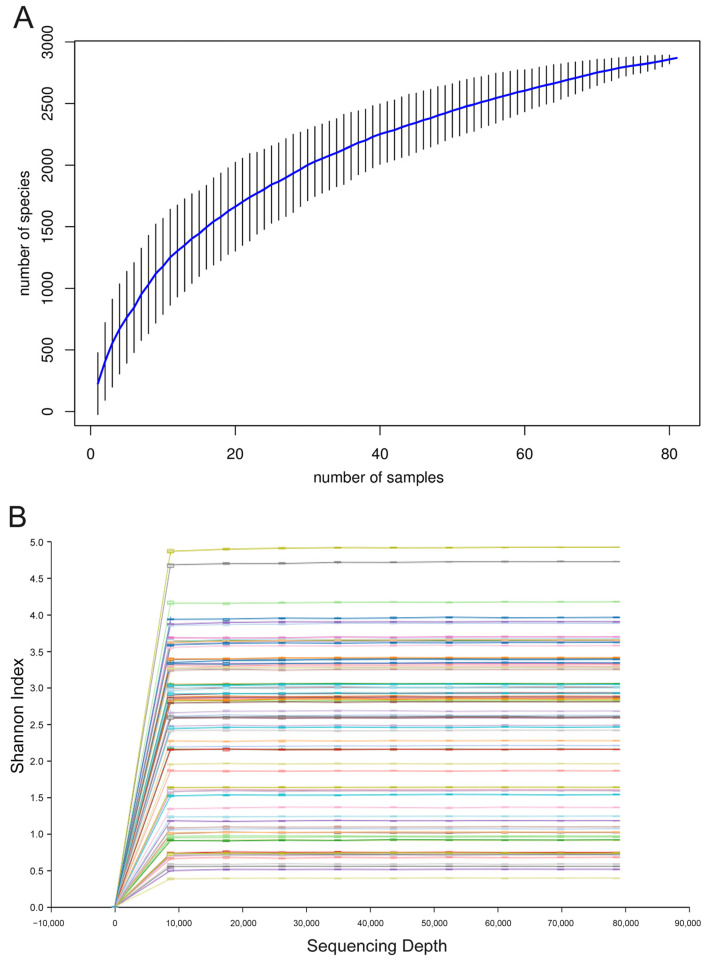
Species accumulation curves and rarefaction curves. (**A**) Species accumulation curves of all the investigated sika deer samples. The horizontal axis represents the number of samples, the vertical axis represents the number of species, and vertical lines show the confidence intervals at various positions along the curve. (**B**) Rarefaction curves of all the investigated sika deer samples. The horizontal axis is the sequencing depth, and the vertical axis is the box plot of the Shannon index. Each line represents an individual fecal sample.

Based on *rbcL* metabarcoding sequencing data, a total of 8,609,690 valid sequences were obtained from all the sika deer fecal samples, with an average of 106,293 ± 14,824 sequences per sample. Species accumulation curves are used to measure and predict the increase in species richness within a community as sample size increases and are widely employed to assess whether the sample size is sufficient and to estimate community richness [39]. The results showed that as the sample size increased, the species accumulation curve gradually leveled off, indicating that the sample size in this study was sufficient to reflect the species composition of the sika deer’s food types (Figure 2A). To assess the diversity and adequacy of sequencing depth, a rarefaction curve was plotted for each sample [40]. The results showed that as sequencing depth increased, the rarefaction curve quickly reached a plateau. This indicates that the alpha diversity has been fully obtained, and the sequencing depth of this study is adequate (Figure 2B).

### 3.2. Relative Abundance of the Diet Composition in C. n. kopschi and C. n. hortulorum

**Figure 3:**
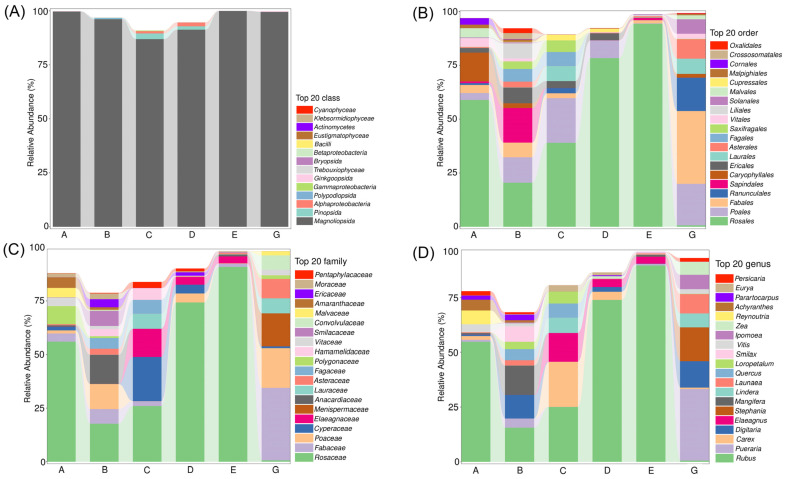
The classes (**A**), orders (**B**), families (**C**), and genera (**D**) with relatively high abundance in the diet composition of *C. n. kopschi* and *C. n. hortulorum*.

**Figure 4:**
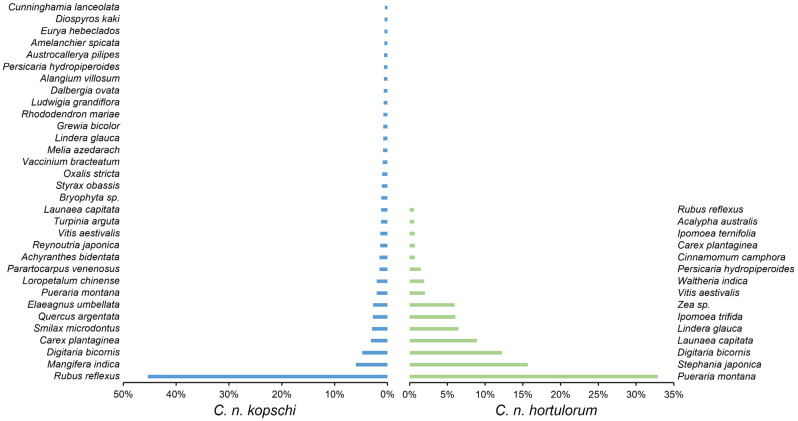
The plant species in the diet composition of *C. n. kopschi* (**left**) and *C. n. hortulorum* (**right**). The *x*-axis represents relative abundance, and the *y*-axis represents plant species.

The classes, orders, families, and genera with the highest relative abundance in the diet composition of *C. n. kopschi* are presented in Table 1 and Figure 3. At the phylum level, all of the diet composition belongs to Streptophyta. At the class level, the dominant class is Magnoliopsida, with a relative abundance of 98.10%, followed by Pinopsida (0.51%) and Polypodiopsida (0.17%) (Figure 3A). At the order level, the dominant order is Rosales, with a relative abundance of 51.26%, followed by Poales (8.72%), Sapindales (7.02%), Ericales (4.06%), and Fabales (3.92%) (Figure 3B). At the family level, the dominant family is Rosaceae, with a relative abundance of 46.73%, followed by Anacardiaceae (6.02%), Poaceae (5.54%), Fabaceae (3.92%), Cyperaceae (3.19%), and Smilacaceae (3.00%) (Figure 3C). At the genus level, the dominant genus is *Rubus*, with a relative abundance of 45.43%, followed by *Mangifera* (5.98%), *Digitaria* (4.77%), *Carex* (3.13%), *Smilax* (3.00%), *Quercus* (2.74%), and *Elaeagnus* (2.74%) (Figure 3D). At the species level, 32 species have a relative abundance greater than 1% (Figure 4). *Rubus reflexus* is the absolute dominant species, with a relative abundance of 45%, followed by *Mangifera indica* (6%) and *Digitaria bicornis* (5%). The next species are *Carex plantaginea*, *Smilax microdontus*, *Quercus argentata*, and *Elaeagnus umbellata*, each with a relative abundance of 3%.

The classes, orders, families, and genera with the highest relative abundance in the diet composition of *C. n. hortulorum* are presented in Table 1 and Figure 3. At the phylum level, all species belong to Streptophyta. At the class level, the main class is Magnoliopsida, with a relative abundance of 99.67%, followed by Ginkgoopsida (0.28%) and Polypodiopsida (0.05%) (Figure 3A). At the order level, the dominant order is Fabales, with a relative abundance of 33.89%, followed by Poales (19.29%), Ranunculales (15.65%), Asterales (8.94%), and Laurales (7.15%) (Figure 3B). At the family level, the dominant family is Fabaceae, with a relative abundance of 33.89%, followed by Poaceae (18.43%), Menispermaceae (15.65%), Asteraceae (8.94%), Lauraceae (7.15%), and Convolvulaceae (6.82%) (Figure 3C). At the genus level, the dominant genus is *Pueraria*, with a relative abundance of 32.87%, followed by *Stephania* (15.65%), *Digitaria* (12.23%), *Launaea* (8.91%), *Ipomoea* (6.73%), *Lindera* (6.45%), and *Zea* (5.96%) (Figure 3D). At the species level, 15 species have a relative abundance greater than 1%, with *Pueraria montana* being the absolute dominant species, accounting for 33%, followed by *Stephania japonica* (16%), *Digitaria bicornis* (12%), and *Launaea capitata* (9%), with *Lindera glauca*, *Ipomoea trifida*, and *Zea sp.* each having a relative abundance of 6% (Figure 4).

### 3.3. Alpha Diversity of the Diet Composition Between C. n. kopschi and C. n. hortulorum

**Figure 5:**
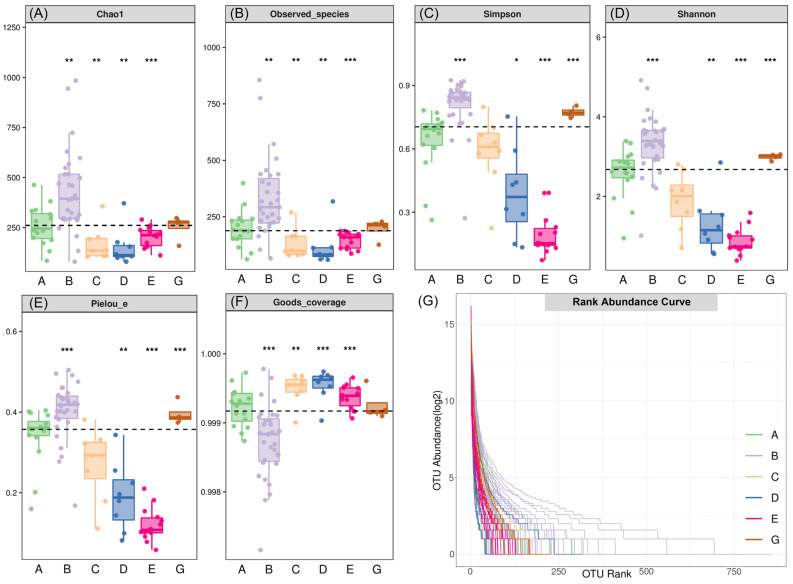
Alpha diversity indices (**A**–**F**) and rank abundance curves (**G**) of the diet compositions in *C. n. kopschi* (Area A–E) and *C. n. hortulorum* (Area G). Each panel corresponds to a specific alpha diversity index, with the gray area at the top indicating the index type. All *p*-values were less than 0.0001. The asterisk represents the difference between the area below it and all other areas. *, **, and *** represent *p*-values less than 0.05, 0.01, and 0.001, respectively.

The alpha diversity of diet composition within different areas of *C. n. kopschi* and *C. n. hortulorum* was comprehensively evaluated using indices such as Chao1, observed species, Shannon, Simpson, Pielou’s evenness, and Good’s coverage. The Chao1 and observed species indices were used to represent richness, reflecting the number of species in a single sample [39]. Among all areas, Area B had the highest richness (Figure 5A, B). For *C. n. kopschi*, Area B had the highest richness, followed by Area A, Area E, and Area C, with Area D having the lowest richness. There were significant differences (*p* < 0.01) between Area B and Areas C, D, and E (Table 2). Although the richness of diet composition in *C. n. hortulorum* (Area G) is lower than that in Area B and higher than that in Area A of *C. n. kopschi*. Overall, there is no significant difference in the richness of diet composition between *C. n. hortulorum* and each area of *C. n. kopschi* (*p* > 0.05).

The diversity was characterized by the Shannon [36] and Simpson [37] indices (Figure 5C,D), reflecting the presence and abundance of species in a community. The evenness was characterized by the Pielou’s evenness index, reflecting the evenness of each species in the community [38] (Figure 5E). Among all areas, Area B exhibited the highest diversity and evenness. For *C. n. kopschi*, the diversity and evenness of Area A, Area C, and Area D closely followed Area B, while Area E had the lowest. There were significant differences (*p* < 0.05) in diversity and evenness between Area B and Areas C, D, and E (Table 2). The differences in diversity and evenness between Area A and Area E were also significant (*p* < 0.05). The diversity and evenness of the diet composition in *C. n. hortulorum* were lower than those in Area B but higher than those in Area A of *C. n. kopschi*. However, the differences were not significant (*p* > 0.05). The diversity and evenness of the diet composition in *C. n. hortulorum* were significantly higher than those in Area E of *C. n. kopschi* (*p* < 0.05).

The coverage rate of each sample library was characterized by the Good’s coverage index, and the higher the value, the lower the probability of sequences in the sample not being detected [40,41] (Figure 5F). Among all areas, Area B had the lowest coverage, which was significantly lower than the coverage of Areas A, C, D, and E (*p* < 0.05, Table 2). There were no significant differences in coverage between any other two areas. The rank abundance curve reflects the distribution pattern of OTU abundance in each sample and can directly describe the species richness and evenness of different community areas. The flatter the curve, the smaller the abundance differences between the OTU in the community, indicating higher evenness in community composition. Conversely, the steeper the curve, the lower the evenness. According to the rank abundance curve (Figure 5G), the species evenness in Area B was significantly higher than in the other areas, while Area E and Area D had the lowest evenness.

### 3.4. Beta Diversity of the Diet Composition Between C. n. kopschi and C. n. hortulorum

**Figure 6:**
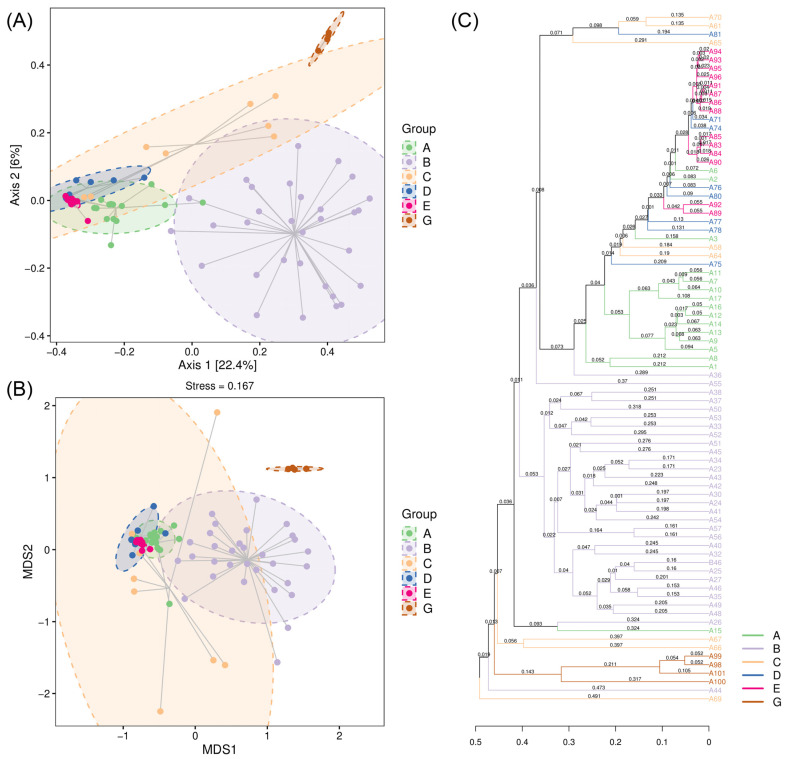
Beta diversity of the diet composition between *C. n. kopschi* (Area A–E) and *C. n. hortulorum* (Area G). (**A**) Principal coordinate analysis (PCoA). (**B**) Nonmetric multidimensional scaling (NMDS). (**C**) UPGMA clustering analysis based on Bray–Curtis distance. The numbers represent the branch distance. The labels on the far right represent the sample.

Principal coordinate analysis (PCoA) showed that the differences in sample composition were explained by PCoA1 and PCoA2, with an explanation of 22.4% and 6%, respectively, resulting in a total explanation of 28.4% (Figure 6A). Nonmetric multidimensional scaling (NMDS) indicated a stress value of 0.167, which is less than 0.2, suggesting that the analysis accurately reflects the degree of difference between the samples (Figure 6B). Based on the results of both PCoA and NMDS, the diet composition of *C. n. kopschi* was clearly separated from that of *C. n. hortulorum*, indicating a significant difference in diet composition between *C. n. kopschi* and *C. n. hortulorum*. For *C. n. kopschi*, the separations between Area B and other areas are more obvious, indicating that there are significant differences in the diet composition of Area B compared to other areas, with the greatest difference between Area B and Areas D and E.

The UPGMA clustering analysis based on the Bray–Curtis distance shows that *C. n. kopschi* and *C. n. hortulorum* are far apart and branch into two major clusters (Figure 6C). For *C. n. kopschi*, Area E and Area D are the closest, forming one branch, then forming one branch with Area A, forming one branch with Area C, and finally forming one branch with Area B.

Based on the Permanova and Anosim algorithms, an analysis of inter-area differences was conducted to test the significance of differences between each area. All areas showed significant differences (*p* < 0.01, Table 3). In addition, non-parametric multivariate ANOVA based on Bray–Curtis distance was used to further analyze the differences between areas. The R^2^ value of Adonis was 0.456 (*p* = 0.001, *p* < 0.01), indicating significant differences between the areas.

### 3.5. Differences in the Diet Composition Between C. n. kopschi and C. n. hortulorum

**Figure 7:**
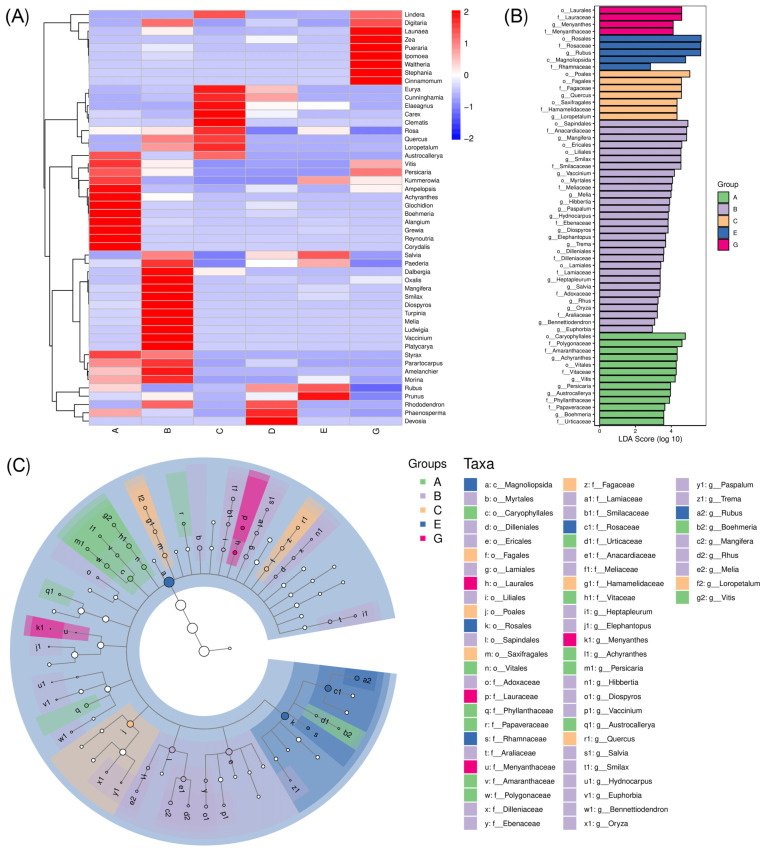
Differences in the diet composition between *C. n. kopschi* (Area A–E) and *C. n. hortulorum* (Area G). (**A**) Species composition heatmap of the diet composition. The values represent abundance, with red and blue blocks representing higher and lower abundance. (**B**) Discriminant diet composition features from LDA test. (**C**) Cladogram of diet composition items from LDA test with concentric circles from inside to outside representing the taxonomic level from phylum to genus. c—class, o—order, f—family, and g—genus.

In order to further compare the differences in species composition between samples and display the distribution trend of species abundance for each sample, we defaulted to using the abundance data of the top 50 genera with the highest average abundance to draw a heatmap for diet composition analysis (Figure 7A). For *C. n. kopschi*, Area A had the highest abundance of *Corydalis*, *Reynoutria*, *Grewia*, *Alangium*, *Boehmeria*, and *Glochidaion*. Area B had the highest abundance of *Dalbergia*, *Oxalis*, *Mangifera*, *Smilax*, *Diospyros*, *Turpinia*, and *Melia*. Area C had the highest abundance of *Eurya*, *Cunninghamia*, *Elaeagnus*, *Carex*, *Clematis*, *Rosa*, and *Quercus*. Area D had the highest abundance of *Devosia* and *Phaenosperma*. Area E had the highest abundance of *Prunus* and *Rubus*. For *C. n. hortulorum* (Area G), *Cinnamomum*, *Stephania*, *Waltheria*, *lpomea*, *Pueraria*, *Zea*, and *Launaea* had the highest abundance.

LDA effect size analysis (LEfSe) was used to search for food species whose relative abundance varied significantly among areas (Figure 7B). In the diet composition of *C. n. hortulorum*, the highest LDA_score is found in Laurales, Lauraceae, *Menyanthes*, and Menyantheceae, indicating that Laurales and Asterales are the dominant orders in the diet composition of *C. n. hortulorum*, especially the Lauraceae family and *Menyanthes* genus in the Menyantheceae family. Compared with *C. n. hortulorum*, eleven higher LDA_scores were found in the diet composition of *C. n. kopschi*, including Caryophyllales and Polygonaceae in Area A; Anacardiaceae, Ericales, Mangifera, and Sapindales in Area B; Poales in Area C; and Rubus, Rosales, Magnoliopsida, and Rosaceae in Area E. In the diet composition of *C. n. kopschi*, the highest LDA_scores are Rosales, Rosaceae, and Rubus, all belonging to Area E, followed by Poales from Area C and Sapindales, Anacardiaceae, and Mangifera from Area B. This indicates that Rosales, Poales, and Sapindales are the dominant orders in the diet composition of *C. n. kopschi*, especially the *Rubus* genus in the Rosaceae family and the *Mangifera* genus in the Anacardiaceae family.

The cladogram is used to display the taxonomic hierarchical distribution of food species in each area of samples, as shown in Figure 7C. In the diet composition of *C. n. hortulorum*, the relative abundance of the Laurales order and Menyantheaceae family is relatively high, which is due to the high relative abundance of the Lauraceae family and *Menyanthes* genus. In the diet composition of *C. n. kopschi*, Area A has a relatively high relative abundance in the Caryophyllales order (*Persicaria* genus in Polygonaceae family and *Achyranthes* genus in Amaranthaceae family), Vitales order (*Vitis* genus in Vitaceae family), Phyllanthaceae family, Papaveraceae family, *Austrochallerya* genus, and Urticaceae family (*Boehmeria* genus). Area B has relatively high relative abundance in the Myrtalales order, Dilleniales order (*Hibbertia* genus in Dilleniaceae family), Ericales order (*Diospyros* genus in Ebenaceae family and *Vaccinium* genus), Lamiales order (*Salvia* genus in Lamiaceae family), Liliales order (*Smilax* genus in Smilacaceae family), Sapindales (*Melia* genus in Meliaceae family, *Rhus* genus in Anacardiaceae family, *Mangifera* genus in Anacardiaceae family), Adoxaceae family, Araliaceae family (*Heptapleurum* genus), Dilleniaceae family (*Hibbertia* genus), and Ebenaceae family (*Diospyros* genus). Area C has a relatively high relative abundance in the Fagales order (*Quercus* genus in Fagaceae family), Saxifragales order (*Loropetalum* genus in Hamamelidaceae family), and Poales order. The relative abundance of the Rosales order (*Rubus* genus in Rosaceae family and Rhamnaceae family) in Area E is relatively high.

## 4. Discussion

Understanding the food preferences of wildlife helps to understand their ecological habits and develop targeted habitat conservation and restoration measures [7,8]. We studied the diversity and differences in diet composition between *C. n. kopschi* and *C. n. hortulorum* in the same distribution area using fecal DNA metabarcoding technology. We analyzed the differences in diet composition of *C. n. kopschi* populations in different distribution areas and gained insight into their dietary habits. This provides an important scientific basis for further developing measures to protect and restore the *C. n. kopschi* population.

The diet composition of *C. n. kopschi* is mainly the Rosaceae family (Rosales order), with a relative abundance of 46.73%. The selection of the Rosaceae family confirms that *C. n. kopschi* seek out more palatable and high-quality food sources. In previous studies on the diet of deer [42,43,44], the Rosaceae family represented the bulk of the diet. In addition, a previous study showed that *C. n. kopschi* distributed in Zhejiang, China consumed a large number of herbaceous plants in summer, with the Poaceae family occurring at a higher frequency in the diet than others. However, in autumn, the proportion of Poaceae family decreased, while the proportion of Fabaceae and Cyperaceae families increased [45]. The sampling time of this study was in summer, and the relative abundance of Poaceae family was indeed higher than that of Fabaceae and Cyperaceae families, confirming the previous study. At the genus level, *Rubus* dominate the diet of *C. n. kopschi*, with a relative abundance of up to 45.43%. Jiang et al. found that *C. n. kopschi* prefer 37 plant species, such as *Smilax china*, *Rubus chingii*, *Rhododendron simsii*, *Rhus chinensis*, and *Cunninghamia lanceolata* [46]. Li et al. found that *Smilax* and *Rubus* are two genera with relatively high abundance in the diet composition of *C. n. kopschi* [42]. Our study is basically consistent with the above research results, as the plants of the *Rubus* genus constitute the main diet composition of *C. n. kopschi*, especially *Rubus reflexius*.

The diet composition of *C. n. hortulorum* are mainly Fabaceae, Poaceae, Menispermaceae, Asteraceae, Lauraceae, and Convolvulaceae, with relatively average plant consumption. At the genus level, the diet composition of *C. n. hortulorum* are mainly *Pueraria*, *Stephania*, and *Digitaria*. *Pueraria montana* are the preferred plant of *C. n. hortulorum*. *C. n. hortulorum* in the study were raised on an enclosed farm within the nature reserve and had a different diet composition from the deer living in northeast China. *C. n. hortulorum* living in northeast China mainly feed on Rosaceae, Betulaceae, Sapindaceae, Urticaceae, and Fagaceae [47], and *Quercus mongolica* was the most commonly consumed plant species [48]. Inappropriate dietary composition and proportion may lead to weight loss and affect the health of captive sika deer [49]. For example, Rosaceae and Leguminosae may need to be added to the feeding menu of *C. n. hortulorum* raised on an enclosed farm within the nature reserve. However, conducting feeding experiments based on local vegetation characteristics and the plant preferences of sika deer may help to accurately obtain the diet composition of *C. n. hortulorum* raised in southern China.

The richness, diversity, and evenness of diet composition in Area B of *C. n. kopschi* are the highest among all areas, and there are significant differences compared to Areas C, D, and E. Area B is located in the experimental area of the nature reserve, with an altitude of 50–100 m. During patrols, the management personnel of the nature reserve often discover a large population of *C. n. kopschi* in this area. This area is a nursery base, where a rich variety of nursery plants are planted, such as *Morus alba*, *Rhus chinensis*, *Prunus persica*, *Ligustrum lucidum*, *Heptapleurum heptaphyllum*, etc. Animals’ selection of habitats with high food abundance is undoubtedly economical [50,51], and the diverse edible plants in Area B may be one of the reasons for this result. However, it is worth noting that the plant species in the core area of the nature reserve are very rich in summer, and the altitude is relatively high, with stricter management and protection measures. For example, Area A, Area C, and Area D are all located in the core area. But *C. n. kopschi* still ventures down the mountain to Area B with lower altitude and frequent human activities, indicating that *C. n. kopschi* may have adopted an ‘adventurous’ feeding strategy for more diverse food resources. In seasons with abundant food, animals usually choose high-nutrient foods to eat, and food may not be the main factor determining animal habitat choices [52,53,54]. Of course, this depends on locations that pose no threat to animal life and contain high-nutrient foods. In fact, there are a large number of human activities around the Taohongling Nature Reserve, such as villages, towns, and even county towns, especially in Area B, which belong to nursery bases with frequent human activities. A prominent feature of wild ungulates is their vigilance toward and avoidance of humans and predators, often choosing habitats with higher levels of concealment and lower disturbance [55,56,57]. We speculate that the long-term ‘crowded’ joint use of natural resources by *C. n. kopschi* and the surrounding community residents in the nature reserve is gradually forming a harmonious coexistence of survival. In addition, the active science popularization and strict patrol management of the nature reserve over the years have also provided protection for the ‘adventurous’ feeding strategy of *C. n. kopschi*. Furthermore, during our investigation, we also found a large number of traces of *C. n. kopschi* activity in Area B, such as gnawing on mulberry leaves, sleeping under peach trees, footprints, etc. There was also a large amount of fresh feces, obviously excreted on the morning or night before our investigation, including adult feces and juvenile feces. These suggest that there is a healthy and stable population of *C. n. kopschi* distributed in Area B, and their feeding activities are separated from human activities in terms of time by choosing the evening or early morning when there is basically no human activity. The phenomenon of separation between the activity time of wild animals and humans is common in high-density human activity areas, and wild animals such as forest elephants (*Loxodonta cyclotis*) [58], black-tailed deer (*Odocoileus hemionus columbianus*) [59], and chimpanzees (*Pan troglodytes schweinfurthii*) usually choose to be active at night [60].

Principal coordinate analysis (PCoA) and nonmetric multidimensional scaling (NMDS) both indicated a significant separation in the diet composition of *C. n. kopschi* and *C. n. hortulorum*, which is related to the supplementary feeding of *C. n. hortulorum* by farms. Although they live in the same area, *C. n. hortulorum* are fed not only with local plants but also with other commercially available plants. There are significant differences in the diet composition between Area B and other areas of *C. n. kopschi*. UPGMA clustering analysis based on Bray–Curtis distance shows that Area E and Area D first cluster together, then cluster together with Area A, then cluster together with Area C, and finally cluster together with Area B. The differences in the distribution pattern of food resources play an important role in the migration of ungulates, and the maximum energy input may be the main driving force for animals to choose habitats [61]. Areas D and E are located on the northern and eastern slopes of the nature reserve, with elevations ranging from 100 to 150 m and similar vegetation types. Area A area belongs to the key distribution area of sika deer in the nature reserve, with a high altitude of 430–470 m. Both Area B and Area C are located on the western slope of the nature reserve. Area C has an altitude between 220 and 270 m and a relatively simple vegetation type, mostly consisting of trees, while Area B is a nursery base with a rich and diverse variety of plant species.

Analysis of species composition differences can display trends in species abundance distribution and identify species differences between areas. Rosales, Poales, and Sapindales are the dominant orders in the diet composition of *C. n. kopschi*, especially the *Rubus* genus in the Rosaceae family and the *Mangifera* genus in the Anacardiaceae family. Many species in the above two genera contain fruits. Previous studies have shown that fruits are the favorite food of deer (such as *Capreolus capreolus*) in summer and early autumn [43]. The differences in relative abundance of diet composition of *C. n. kopschi* have been observed in different geographical areas, which are closely related to the plant characteristics of the different geographical areas. Similar findings have also been found in *C. n. yakushimae* in Yakushima Island, Japan [62]. Area A is an experimental area for forest dwarfing in the habitat of deer within the nature reserve, which is a protective measure taken by the nature reserve to protect the habitat of the deer. The dietary composition in this area is mainly composed of shrubs and herbs, such as *Persicaria*, *Achyranthes*, and *Boehmeria* genera. Area B is a nursery planting base, where *C. n. kopschi* feed on relatively more cultivated plants such as *Smilax*, *Heptapleurum*, and *Diospyros*.

DNA metabarcoding has become an increasingly popular tool for monitoring animal diet [63,64,65,66]. The read counts of DNA metabarcoding are extremely dependent on the amount of DNA as well as the number of gene copies and primer bias [67,68], but it can still provide a preliminary or semi-quantitative estimation of relative abundance [69]. Although the relative abundance of reads is not an accurate measure of abundance at the community level, we assume that these biases will not have a significant impact on intra-taxon comparisons between samples [70]. Therefore, these data can be used to examine the comparative differences in the identified taxa of diet composition between *C. n. kopschi* and *C. n. kopschi*. In future research, multiple genetic markers should be used to increase the probability of species detection and accuracy of species quantification. Additionally, further studies are needed to valuate occurrence in the diet relative to availability in the environment, such as direct observation method, utilization method, fecal microscopic analysis method, and feeding experiments, as well as to establish analysis models of diet composition and plant species to further understand the relationship between the diet and habitat of sika deer.

## 5. Conclusions

This study investigated the diet composition of South China sika deer (*Cervus nippon kopschi*) based on DNA metabarcoding technology. The dominant family and genus in the diet composition of *C. n. kopschi* were Rosaceae and *Rubus*, respectively. Thirty-two preferred plant species were identified in *C. n. kopschi*, and the highest relative abundance was *Rubus reflexus*. The dominant family and genus in the diet composition of *C. n. hortulorum* were Fabaceae and *Pueraria*, respectively. Of the 15 preferred plant species, the highest relative abundance was *Pueraria montana*. Our findings further elaborate on diet composition differences among different areas of *C. n. kopschi* and *C. n. hortulorum* raised in the same area. This study elucidates the diet composition of sika deer and provides reference for the protection and improvement of South China sika deer habitats of Taohongling Sika Deer National Nature Reserve. However, further studies are required to determine the food preferences and differences of South China sika deer in different seasons on a larger scale.

## Figures and Tables

**Table 1:** Top 10 orders, families, and genera with the highest relative abundance (RA) in the diet composition of *C. n. kopschi* and *C. n. hortulorum*.

Rank	*C. n. kopschi*						*C. n. hortulorum*					
	Order	RA (%)	Family	RA (%)	Genus	RA (%)	Order	RA (%)	Family	RA (%)	Genus	RA (%)
1	Rosales	51.26	Rosaceae	46.73	*Rubus*	45.43	Fabales	33.89	Fabaceae	33.89	*Pueraria*	32.87
2	Poales	8.72	Anacardiaceae	6.02	*Mangifera*	5.98	Poales	19.29	Poaceae	18.43	*Stephania*	15.65
3	Sapindales	7.02	Poaceae	5.54	*Digitaria*	4.77	Ranunculales	15.65	Menispermaceae	15.65	*Digitaria*	12.23
4	Ericales	4.06	Fabaceae	3.92	*Carex*	3.13	Asterales	8.94	Asteraceae	8.94	*Launaea*	8.91
5	Fabales	3.92	Cyperaceae	3.19	*Smilax*	3.00	Laurales	7.15	Lauraceae	7.15	*Ipomoea*	6.73
6	Caryophyllales	3.76	Smilacaceae	3.00	*Quercus*	2.74	Solanales	6.82	Convolvulaceae	6.82	*Lindera*	6.45
7	Fagales	3.17	Fagaceae	2.76	*Elaeagnus*	2.74	Vitales	2.26	Vitaceae	2.26	*Zea*	5.96
8	Liliales	3.00	Elaeagnaceae	2.74	*Pueraria*	2.05	Malvales	1.94	Malvaceae	1.94	*Vitis*	2.03
9	Saxifragales	2.06	Polygonaceae	2.16	*Loropetalum*	2.04	Caryophyllales	1.75	Polygonaceae	1.75	*Waltheria*	1.94
10	Vitales	1.48	Hamamelidaceae	2.04	*Parartocarpus*	1.50	Rosales	0.68	Cyperaceae	0.87	*Persicaria*	1.73

**Table 2:** Pairwise comparisons (*p*-values) of the Alpha diversity index between different areas of sika deer.

Comparison	Chao1	Observed Species	Simpson	Shannon	Pielou’s Evenness	Good’s Coverage
A-B	0.0669	0.0876	0.0077	0.0915	0.1216	0.0469
A-C	0.6217	0.7745	1.0000	0.8564	0.9988	0.6117
A-D	0.2809	0.2492	0.8443	0.1887	0.1887	0.3210
A-E	1.0000	1.0000	0.0133	0.0047	0.0025	1.0000
A-G	1.0000	1.0000	1.0000	1.0000	1.0000	1.0000
B-C	0.0004	0.0008	0.0133	0.0052	0.0100	0.0003
B-D	0.0001	0.0001	0.0002	0.0001	0.0002	0.0001
B-E	0.0007	0.0002	0.0000	0.0000	0.0000	0.0008
B-G	0.9935	1.0000	1.0000	1.0000	1.0000	0.6088
C-D	1.0000	1.0000	1.0000	1.0000	1.0000	1.0000
C-E	1.0000	1.0000	0.2540	0.6491	0.4106	1.0000
C-G	1.0000	1.0000	0.8443	0.5789	0.4100	1.0000
D-E	1.0000	1.0000	1.0000	1.0000	1.0000	1.0000
D-G	0.9935	0.7745	0.2479	0.1607	0.0972	1.0000
E-G	1.0000	1.0000	0.0128	0.0192	0.0063	1.0000

Note: *p*-values less than 0.05 were considered significant.

**Table 3:** Significant analysis of inter-area differences in diet composition of sika deer in different areas based on Permanova and Anosim algorithms.

Comparison	Permanova		Anosim	
	Pseudo-F	*p*-Value	R^2^	*p*-Value
A-B	17.979	0.001	0.556	0.001
A-C	7.321	0.001	0.631	0.002
A-D	5.291	0.002	0.361	0.002
A-E	18.098	0.001	0.538	0.001
A-G	25.430	0.001	1.000	0.003
B-C	6.182	0.001	0.721	0.001
B-D	11.770	0.001	0.574	0.001
B-E	25.222	0.001	0.615	0.001
B-G	6.558	0.001	0.721	0.001
C-D	3.879	0.002	0.281	0.008
C-E	11.895	0.001	0.725	0.001
C-G	5.541	0.004	0.654	0.003
D-E	2.950	0.006	0.483	0.002
D-G	23.373	0.006	1.000	0.001
E-G	106.586	0.001	1.000	0.002

## Data Availability

The data presented in this study are available on request from the corresponding author.
